# Two Distinctions That Help to Chart the Interplay Between Conscious and Unconscious Volition

**DOI:** 10.3389/fpsyg.2019.00552

**Published:** 2019-03-26

**Authors:** Marc Slors

**Affiliations:** Section Philosophy of Mind and Language, Faculty of Philosophy, Theology and Religious Studies, Radboud University, Nijmegen, Netherlands

**Keywords:** conscious intentions, intentional action, mental causation, conscious agency, volition, self-initiated action

## Abstract

Research initiated by Benjamin Libet suggests that short-term conscious intentions are not the onsets of bodily actions. However, other research, particularly on longer-term intentions, seems to show that at least some conscious intentions are effective. This leads to the idea that volition is a complex interplay between conscious and unconscious processes. The nature and structure of this interplay is mostly uncharted territory. In this article, I will highlight two currently neglected distinctions that will help to chart the territory. The first distinction is between intentions we become conscious of (passive) and consciously formed intentions (active). The second is Fred Dretske’s distinction between structuring and triggering causes. I will introduce both distinctions by discussing how they tie in with and strengthen recent criticism of free selection paradigms and support the idea that consciously self-initiated action issues from processes of conscious deliberation and/or information integration. I will argue that consciously self-initiated action typically involves consciously formed intentions that are the structuring causes of our actions. This notion of conscious intentional action allows us to identify at least four stages in which unconscious processes co-determine our actions—without undermining their self-initiated character.

## Introduction

Do conscious intentions cause behavior? There is an impressive literature, based on extensive experimental evidence, suggesting that our actions are not, in fact, initiated by conscious intentions in the way it is often claimed we experience this to be the case (Libet et al., 1983; Haggard and Eimer, 1999; Libet, 1999; Wegner, 2002; Brass and Haggard, 2008; Soon et al., 2008; Fried et al., 2011). There is a less extensive, but just as interesting literature, also based on solid experimentation, however, according to which some conscious intentions *are* the causes of some of our actions (Webb and Sheeran, 2006; Baumeister and Masicampo, 2010; Baumeister et al., 2011). Research on implementation intentions (Gollwitzer, 1999) and research on the effects of consciously anticipated regret (Janis and Mann, 1977; Tetlock and Boettger, 1994; Richard et al., 1996) are cases in point. There need not be a contradiction between these bodies of literature because the experiments cited in both target different kinds of intentions. Roughly speaking, the non-efficacious intentions are to be found in the realm of intentions that occur directly before acting, while the effective ones are often longer-term intentions. Thus, those in favor of effective conscious intentions agree that “consciousness may be ill suited for direct control of physical behavior” (Baumeister et al., 2011, p. 352). According to them, “conscious causation is often indirect and delayed, and it depends on interplay with unconscious processes” (Baumeister et al., 2011, p. 331; see also Baumeister and Masicampo, 2010, p. 948).

This more nuanced picture of human motivation as an interplay between conscious and non-conscious processes is arguably a step forward, compared to the simple affirmation or rejection of the efficacy of conscious intentions. Not much has been done, however, to fill in this more complex picture of human motivation and to chart the ways in which conscious and unconscious processes connect and collaborate to produce what is known as “self-initiated” action. In this article, I want to make a case for the claim that this is in part due to a lack of necessary conceptual distinctions. I will introduce two distinctions pertaining to the main concepts at play–“conscious intention” and “causing behavior”–and show how they can help to draw a more nuanced picture of human volition.

In order to do this, I will take what may seem like a detour. I will introduce the two distinctions *via* relatively recent philosophical and scientific criticism of “free selection” paradigms–of which Libet’s experiments are the best known example–as paradigmatic instances of free will or, more neutrally formulated, consciously self-initiated action. This discussion will set the stage for a picture of conscious volition in which unconscious processes play a crucial part in at least four stages of the process of forming an intention and acting on it. In a bit more detail, the plan of this article is as follows:

In Free Selection Versus Conscious Deliberation, I will discuss existing reservations, both from philosophy and from neuroscience, about the relevance of and implicit assumptions behind free selection paradigms and the call for more research on self-initiated actions conceived of as actions that follow from deliberation or complex integration of information. Part of this criticism pertains to the interpretation of Libet-style experiments as experiments about freedom of the will. In the remainder of this article, I will not be concerned with free will but with consciously self-initiated actions only. In Two Kinds of Conscious Intention, I will introduce a distinction between (1) intentions we become conscious of and (2) consciously formed. I will argue that this distinction ties in with the call for a shift in research focus discussed in Free Selection Versus Conscious Deliberation and strengthens it considerably.

In External Influences, Self-Initiation, and Two Types of Causation, I will discuss the extent to which consciously self-initiated action can involve external cues or causes. Adherents of free selection paradigms typically think that this is not the case, but their critics think that external cues and causes are unavoidable. This raises the question which external cues and causes do and which do not undermine the self-initiated character of actions. I will argue that Fred Dretske’s distinction between triggering and structuring causes helps to answer this question.

The upshot of the discussion of these issues will be that the kinds of conscious intentions involved in typical instances of consciously self-initiated action are consciously formed intentions that are the structuring causes of behavior. In The Interplay Between Conscious and Unconscious Processes in Consciously Self-Initiated Action, I will argue that this type of conscious volition allows for and often requires interplay with unconscious processes at four different stages of the unfolding of a willed action–without undermining their consciously self-initiated character.

## Free Selection Versus Conscious Deliberation

The 1983 experiment by Benjamin Libet and colleagues is often credited as being the start of the experimental, neuro-scientific approach to the problem of free will. As is well known, Libet found that a conscious intention to lift a finger at any time the subject chooses was preceded by an unconscious readiness potential (Kornhuber and Deecke, 1965) that is most often interpreted as the actual onset of the finger movement (see, however, Mele, 2006; Miller et al., 2011; Schurger et al., 2012). This finding has been replicated several times, not just using EEG, but also using fMRI (Soon et al., 2008) or electrodes in the brain (Fried et al., 2011). Moreover, an unconscious readiness potential is found to precede and predict not only a “when” choice but also a (very limited) “what” choice [do I press the left or the right button? (Haggard and Eimer, 1999)] or even a “whether” choice [do I press a button at all? (Brass and Haggard, 2008)]. These experiments have been and still are very influential. They play a large role in Daniel Wegner’s more “grand scale” attack on the illusion of conscious will (Wegner, 2002) and persuade many that we do not have free will (Libet, 1999). Critique on Libet-style experiments from within neuroscience and psychology has focused on methodological issues and/or on the trivial or artificial nature of the task of finger-lifting as a paradigmatic case for freedom of the will. More recent (or better: more recently published) critique pertains not so much to the trivial or artificial nature of the choice to lift a finger or press a left or right button, but to the groundlessness of such choices.

The type of task studied in Libet-style experiments is known as a free selection task. The choice a subject has to make–in this case: when to lift a finger, or which button to press–is typically a spontaneous one, that is, one about which we cannot really consciously deliberate. At some point, we just decide to do it. As Markus Schlosser analyzes in detail, this is the reason why many philosophers reject the idea that Libet-style experiments are about free will (Schlosser, 2014). It is “widely agreed within the philosophical debate that questions concerning free will are, first and foremost, questions concerning choices and actions that are *based on reasons*. On most views, this does not mean that the relevant choices and actions must be based on conscious deliberation about pros and cons, and it does not mean that they must be based on normatively good reasons. Rather, to say that the relevant choices and actions are based on reasons means, more modestly, that they can be *rationalized* from the agent’s point of view (…)” (Schlosser, 2014, p. 250). This does not mean that “being based on reasons” is generally considered to be sufficient for an action to be free. Many philosophers consider the indeterminacy of the outcome of choices to play an equally important role. Schlosser’s point, however, is that while Libet-style experiments highlight the indeterminacy of choice outcome, many philosophers interested in free will ignore Libet because free selection tasks do not involve acting for a reason, which they consider to be a necessary condition for free will. The complaint that Libet does not address real free will cannot be found in print often (see, however, Dennett, 1991; Mecacci and Haselager, 2015; Asma, 2019). But the rather minimal effect that Libet-style experiments have had on the rich, lively and generally well-informed philosophical debate on free will is a form of criticism in itself.

The kind of freedom that is associated with free selection tasks is usually referred to as “liberty of indifference” in philosophy, not “free will.” According to Schlosser, this does not mean that free selection tasks do not target a specific kind of freedom. They do. The point is rather that this kind of freedom is “insignificant and uninteresting” (Schlosser, 2014, p. 251). Choices that are not based on reasons, i.e., choices that are not grounded in the fact that some feature, consequence, or aspect of the action is “wanted, desired, prized, held dear, thought dutiful, beneficial, obligatory or agreeable” (Davidson, 1963, p. 685) by the agent, are not worth worrying about. This is why Schlosser concludes that research on, e.g., decision making provides us with much more valuable materials to think about free will.

At this point, however, we could distinguish between the practical concerns of people that philosophers are interested in and the theoretical concerns of scientists. Some scientists are interested in the question whether conscious intentions initiate actions. It would avoid confusion and unnecessary polemics when we say that Libet’s research shows that in his experiments there is no “consciously self-initiated action” (despite a phenomenology that seems to tell us otherwise), rather than no “free will.” This description would also fit the purpose of the present paper better. And yet, this terminological change does not mean that free selection advocates are off the hook.

Friederike Schüur and Patrick Haggard identify a problem for free selection tasks as a typical model for self-initiated actions (Schüür and Haggard, 2011). First, they distinguish self-initiated actions in free selection paradigms from other types of self-initiated action found in the literature. In many psychological studies, self-initiated actions are taken to be operant actions that are not triggered by immediate external cues but by internal cues, such as a subject’s own preceding actions, elapsed time, or (changed) behavioral goals. Typically, these internal triggers can be manipulated and controlled in psychological experiments. This is what they refer to as type I self-initiated action. Free selection experiments target what they call type II self-initiated actions. Like type I self-initiated actions, these are not triggered by external cues. But unlike tasks that elicit type I actions, free selection tasks are deliberately designed such that no internal cues can play a role either. The point is, again, that subjects have no specific preferences about when to lift a finger or about pressing a right or a left button and that they cannot really deliberate about this–there are no *reasons* for pressing left or right or lifting a finger now rather than later. At some point, subjects just become conscious of the fact that they have an intention to act. In this sense, type II self-initiated actions are “underdetermined.”

And yet, such actions have to be determined by something. The implicit assumption in the concept of type II self-initiated actions, Schüür and Haggard analyze, is that rather than triggered by internal or external cues, such actions are thought to be issued by what they call an “agential self.” Like Wegner and others, they consider this notion to be derived from subjective experience; we experience ourselves (our conscious selves) to be the authors of our actions. And just like Wegner, they argue that such experience is a poor guide for science. The very notion of an agential self, they argue, is problematic for several reasons. Unanswered scientific questions–What is it? How does it cause behavior? How can it be manipulated in experiments (and if it can be manipulated, do such manipulations not simply count as internal cues)?–are among the less profound ones. The really deep problem is that the agential self plays the role of an Aristotelean “prime mover unmoved,” or an entity that can initiate actions “spontaneously” (Brembs, 2010). Like the familiar homunculus fallacy, this just defers explanation of the origin of actions or at best invites an infinite regress.

Type I self-initiated actions do not exhaust all self-initiated actions, then, but type II is problematic. This is why Schüür and Haggard (2011), p. 1,702 propose a type III self-initiated action, which they define as “the motor consequences of processing and integrating large numbers of qualitatively different types of input”. This “complex integration” may look like an odd definition for self-generated action. The authors themselves even characterize it as less than fully intuitive. But they might not be doing justice to their own proposal. They way in which subjects integrate complex information–i.e., information from different times, locations about different objects and attributes–is sufficiently biography- and character-dependent to yield different, idiosyncratic, personalized responses from different people to the same inputs to count as “self-initiated.” The good move that is made here is that instead of conceiving self-generated actions as “underdetermined,” or “spontaneous,” they are conceived of as being determined by a complex of factors the *integration* of which is highly personal–arguably, this integration is the personal, “self”-factor. *This* “self,” however, is not an Aristotelian unmoved mover, but something that is present in the idiosyncratic–or authentic, if you will–nature of a person’s responses to immediate cues, internal and external.

To study self-initiated action that is not operant, then, we should not rely on free selection paradigms, but rather look at studies on, for instance, decision making. Schüür and Haggard (2011), p. 1,702 claim that studies in decision making such as these are conducted in economics target a specific sub-set of type III self-initiated actions. But they do emphasize that such studies use normative models–models about the most appropriate or correct way of responding–that certainly do not apply in all instances of type III self-initiated action. What is striking, though, is that Schüür and Haggard’s conclusion about how to study self-initiated action and how not resembles Schlosser’s conclusion about how to study free will. Rather than looking at free selection tasks, all agree that we should look at the processes through which people come to their actions summarized under the headings of “deliberation” and “complex integration.” Such processes cover not only decision making, but also more associative or emotional processes of weighing options and less transparent, unconscious processes in which diverse inputs are integrated to yield specific behavior.

Here it might seem as if we are losing sight of the role of consciousness in the production of actions. To some extent that is true. Since we are concerned in this article with the role of consciousness in the production of actions, let us limit our attention to the subset of conscious processes that underlie acting for reasons and complex integration. With respect to consciously self-initiated actions, then, the upshot of the discussion of Schlosser and Schüür and Haggard is that a case can be made for the claim that free selection tasks will largely be uninformative; if we want to study consciously self-initiated action, we should concentrate on conscious decision making, conscious deliberation, and other forms of conscious integration of diverse inputs.

This is not to say that this case is decisive; our discussion so far is too brief for that. In the next section, however, I want to strengthen the criticism of free selection paradigms and the call for research on deliberation and decision making by means of a commonsensical but essential distinction between two types of conscious intention.

## Two Kinds of Conscious Intention

Let me start by introducing, discussing, and defending a distinction between two types of conscious intention. I will then tie this distinction into the discussion on free selection versus conscious deliberation/complex integration and argue that it augments and boosts the case against free selection and for conscious deliberation/complex integration as a paradigm for consciously self-initiated action.

In most of the literature, distinctions between kinds of intentions are limited to temporal distinctions–between long-term or distal intentions, short-term or proximal intentions and motor intentions–or, rarely, differences pertaining to their initiating or monitoring function (Searle, 1983; Brand, 1984; Mele, 1992; Pacherie, 2006). Apart from such temporal/functional differences, conscious intentions, *as such*, are treated as a more or less homogeneous group. This might be due to the association between consciousness and the self (inherited from Descartes). Intentions that are conscious are treated, implicitly or explicitly, as intentions that subjects most clearly consider to be their own. If so, being consciously entertained is a crucially important characterizing feature of intentions. In fact, the association between self and consciousness is what Libet-style experiments implicitly draw on when interpreted as arguments against free will. If we define free will as involving agential control over actions, the argument goes as follows: (1) conscious intentions are *my* intentions, (2) conscious intentions do not control my actions, at best they go along for a ride that is determined by unconscious processes, ergo: (3) *I* do not control my actions. This style of thinking makes being consciously entertained a cardinally important feature of intentions. Thus, it seems natural to treat conscious intentions as one particular class of intentions; further distinctions within that class would not pertain to the fact or the way in which they are conscious.

This, I claim, is a mistake. They way in which intentions are conscious and/or the role of consciousness vis-a-vis their formation can be so different that it makes sense to distinguish between at least two different types of conscious intention. It seems perfectly commonsensical to distinguish between intentions we become or are conscious *of* on the one hand, and intentions that are consciously *formed* on the other. This is the functional difference between *passively registering* an intention versus *actively producing* one. It is likely that the kinds of consciousness involved in these different types are not the same–passively registering probably involves a kind of perceptual or phenomenal consciousness, whereas active formation requires reflective consciousness (Roessler and Eilan, 2003)–but I will not pursue this point here. Instead, I will concentrate on the functional difference.

Consciously registered intentions are those intentions that we become aware of while not being aware of the process leading up to them. To say that they are *passively* registered does not mean that these intentions just pop up; it is to say that consciousness is passive or perceptive relative to these intentions and that their active production is unconscious. They can be intentions we register only after we have started to act on them, like in Searle’s “intentions in action” (Searle, 1983). But awareness of a passively registered intention can also precede action. Suppose you are going to get ice cream, but it is only at the moment that the shopkeeper asks what flavor you would like that you realize that you are craving banana ice cream. At that moment, you become aware *of*–you consciously register–an intention to buy banana ice cream, which in turn determines your order. Intentions we consciously register, passively, are probably a more common phenomenon than the classic Cartesian image of consciously controlled action suggests.

Here a problem might seem to arise. Intentions are executive states. Mele (2006, 2009) has argued that in Libet-style experiments we do not become conscious of such executive states, but merely of an urge to act. This urge is then either given a conscious nod of approval–an avowal–and turned into an intention or it is vetoed. This proposal would give consciousness an active role in producing intentions that I argue are merely passively registered.^1^ The problem with Mele’s move, however, is that it requires a causal link between a conscious intention as an executive state and an action. While Libet allows for such a causal link, other researchers, most notable Daniel Wegner (2002), have argued that actions that follow a conscious intention are in fact caused by unconscious processes associated with the unconscious causes of the intention, thus severing the causal link between a conscious intention and the ensuing action. For this reason, I would favor a notion of the required avowal that is less vulnerable to criticism. A plausible candidate would be to say that an avowal consists in being prepared and able to explain and defend acting on an intention when questioned. This allows for the possibility of intentions we passively become conscious of, *as executive states*. Suppose you are looking for a new place to live and have narrowed your choice down to three houses you have seen. You tell yourself that you need to sleep over it because this kind of decision should not be taken in a few minutes. At some point, however, you can just notice that you have already settled on a particular house. Or suppose you are doubting whether to buy that new car. At some point, you may just notice that your nagging doubt is a signal of the fact that you decided it is too expensive and you should not buy it. This notion of avowing can acknowledge the intuition Mele builds on, that is, the intuition that we sometimes consciously turn an urge into an intention. The point is, though, that it would remain a possibility that the conscious avowal is an ineffective consequence of an effective unconscious avowal.

Actively, consciously produced intentions, by contrast, involve conscious activity that brings intentions into existence. Consciously bringing about an intention is not some magical process through which an intention pops up out of nothing through some conscious “oomph” (to use Wegner’s evocative term). Rather, it involves pondering, weighing of options, decision making, thinking, or generally put, making up one’s mind. Think of forming the intention to have your next vacation in France. This is in all likelihood a conscious process in which you gather information about destinations, preferences of family members, etc. and then decide based on weighing various factors. Or think of deciding what to pick from a restaurant menu. What makes it plausible that consciousness is indeed a necessary part of the formation of such intentions is the fact that their formation requires information that is consciously accessed. Gathering information about holiday destinations or being aware of the various options on a restaurant menu are integral parts of the process of forming an intention, parts without which the intention could not be formed.

To say that consciously gathering information, consciously probing ones preferences, consciously weighing pros and cons, etc. are necessary for the formation of some intentions is not to say that it is sufficient or that every aspect of the process of the formation of such intentions is conscious.^2^ Many decision processes are such that consciously tallying pros and cons, for instance, will not deliver a decisive answer on what to do. Our choices are not fully determined by our conscious beliefs and desires (for if they are, they would not be choices, but simply action intentions that logically follow from and are hence given by our beliefs and desires). What, then, tips the balance in favor of a specific choice? Holton (2009, pp. 53–69) has argued that unconscious preferences and processes attract us to one of the options we can choose from, even though at a conscious level all options might seem equally defensible. This view seems to have both the science of motivation and the phenomenology of choosing on its side (see also The Interplay Between Conscious and Unconscious Processes in Consciously Self-Initiated Action). So, it is not the actual formation of intentions unconscious after all? I would argue that this would be a misleading conclusion. For even when unconscious preferences and processes do play an essential role in forming an intention, the crucial difference between intentions we become conscious of and consciously formed intentions is that in the latter case consciousness plays an ineliminable role–these intentions could not have been produced without the essential contribution of consciousness.

Consciousness can provide what Vierkant (2018), following Hieronymi (2009), calls “managerial control” of deliberation processes leading up to a choice/intention formation. That is, control aimed at facilitating and enabling the process of intention formation, even when part of that process is unconscious. Conscious intentions to choose–that is, intentions to form an intention–can initiate a process of deliberation, for instance (see Wu, 2013). But conscious intentional processes play a role too in terminating deliberation. Shepherd, for instance, argues “direct control over decisions is an extension of the skilled mental activity of deliberation, necessarily involves attention, and is initiated in response to attention-mediated indication that terminating deliberation by forming some intention is appropriate (Shepherd, 2014, pp. 349–350). Furthermore, Vierkant (2018, p. 7) argues that refraining from reopening deliberation after forming an intention that is co-caused by unconscious preferences is also part of the managerial control of deliberation. There is a fourth function of consciousness that makes it a necessary part of some processes leading up to intention-formation and that may also count as contributing to managerial control. One of the most widely accepted features of consciousness in the neuro-scientific literature is that it “has been seen by almost all theorists as helping to integrate information” (Baumeister and Masicampo, 2010, p. 949). Morsella refers to a much longer list of theories on the function of consciousness when he speaks of “the integration consensus.” The idea behind this consensus is that conscious processes “integrate neural activities and information processing structures that would otherwise be independent (…). Many of these theories speak of a central information exchange, where dominant information is distributed globally” (Morsella, 2005, pp. 1001–1002). This is exactly what is required when one is consciously making up one’s mind. Integration, in a metaphor, comes down to being able to “lay all options on the table,” so that we can compare, deliberate, and finally decide and form an intention.

Despite these different types of conscious managerial control over processes of deliberation, it may well be that ultimately an unconscious preference tilts the balance and determines the formation of an intention, as Holton argues. But this does not make the intention an unconsciously produced one. For without the conscious initiation of the deliberation process, consciously laying all options on the table, and the consciously initiated and maintained termination of deliberation by reaching a decision, these unconscious preferences would not have given rise to intention-formation.

Now that the distinction between intentions we become conscious of and intentions we consciously form is in place, we can return to the discussion of the previous section. The first thing to note is that the picture of consciously formed intentions in terms of the information-integrating function of consciousness lines up perfectly with Schüür and Haggard’s views on self-initiated action as requiring what they call “complex integration.” It also fits with the philosophical consensus highlighted by Schlosser that we should focus on intentions that are the product of reasons (although Schlosser does argue that not all such reasons need not be the product of conscious deliberation). The terminology in these three areas of the literature–on the function of consciousness, on the neuroscience of self-initiated actions, and on the science and philosophy of free will–is slightly different, but the ideas seem to overlap. Thus, I take the plea for more research on conscious decision making, complex integration, and conscious deliberation discussed in the previous section as a plea for the idea that paradigmatic instances of consciously self-initiated action are cases of the active conscious formation of an intention to act.

Second, and at least as importantly, the distinction between the two types of conscious intention allows us to better articulate the problem with free selection paradigms. For Schlosser, the main problem is that choices made in such paradigms are uninteresting because they do not involve reasons and deliberation. Schüür and Haggard see another problem, namely the implicit appeal to an “agential self” that appears to function somewhat like an unmoved mover. Both observations can be combined in a single complaint when we use the conceptual distinction between the two types of conscious intention. The combined complaint runs as follows:

Free selection paradigms are presented as if they *might* allow for the conscious formation of intentions, in which case the unconscious initiation of actions that is actually found in such experiments would be a strike against our self-image as conscious agents. If free selection tasks might involve actively consciously formed intentions, however, then there has to be a conscious process by means of which this intention is produced. This process cannot be a process of deliberation or complex integration. There is nothing to deliberate about–there are no reasons for lifting a finger now or later. The implicit idea at play, then, is that there is some conscious “oomph” that can produce intentions just like that. This is what Schüür and Haggard refer to as the idea of an “agential self” that is based on our subjective experience of ourselves. Allegedly, we experience ourselves as consciously *producing* an intention in free selection tasks. But this is the mistake made by Libet and his followers: the fact that we do not experience the process leading up to a conscious intention does not mean that we experience ourselves producing it spontaneously (at best we can–mistakenly–interpret ourselves that way). All we experience is an intention “popping up” in consciousness.

If we stick to the distinction between the two types of conscious intention introduced in this section, we would have to conclude that intentions in free selection tasks are in fact intentions that subjects simply become conscious *of*–*passively*. The intention to lift a finger in Libet’s experiment is like the intention to order banana ice cream that just pops up when we are asked what flavor we prefer. For such intentions, there is no preceding conscious process of deliberation. Yet, as Schüür and Haggard argue, they would have to come from somewhere. Intentions that we passively become conscious of have to be formed, by their very nature, “outside” of consciousness. Therefore, free selection tasks, choosing without being able to deliberate, *must* involve intentions that are formed unconsciously. Thus, Libet et al.’s findings should not have been a surprise. More importantly, free selection paradigms should not count as targeting consciously self-initiated action in the first place.

The upshot of the discussion of Free Selection Versus Conscious Deliberation and Free Selection Versus Conscious Deliberation, then, is that when it comes to consciously self-initiated action, intentions formed through conscious deliberation or conscious integration are the only relevant ones. This is what I will take as point of departure for the rest of this article.

## External Influences, Self-Initiation, and Two Types of Causation

One of the more central differences between intentions that are consciously formed through deliberation, or more generally, through “complex integration” of diverse inputs on the one hand, and the kind of “spontaneously formed” conscious intentions that Libet implicitly supposed are at play in free selection tasks, is that the latter, but not the former, are supposed to be completely free of influences that originate from outside the acting subject. According to Schlosser, Libet holds that “free choices have exclusively internal causes, and free choices are thereby contrasted, implicitly, with choices that are influenced by external factors” (Schlosser, 2014, p. 253). Schüür and Haggard agree, at least partially. Like Schlosser, they believe that Libet wants to exclude external causes from self-initiated actions. Unlike Schlosser, however, as outlined in Free Selection Versus Conscious Deliberation, they believe Libet wanted to exclude internal causes and cues just as well in favor of an “agential self” as the sole source of action: “The reasoning underlying free selection paradigms seems to be that if the degree of self-generatedness of an action is defined by the absence of specification by inputs, then free selection paradigms allow the study of self-generated actions in their ‘purest’ form” (Schüür and Haggard, 2011, p. 1,699). It is not entirely clear whether there is real disagreement here over the role of internal causes in Libet’s notion of free choice, as choices that are (putatively) caused by an agential self might also be thought of as internally caused. Be this as it may, my concern in this section is with external factors that influence self-initiated actions.

In their opposition to the idea that free selection is paradigmatic for self-initiated action, Schüür and Haggard explicitly state that self-initiated actions can and do involve external influences (see also Nachev and Husain, 2010; Ohbi, 2012). According to the “complex integration” account of self-initiated action, “one can correctly claim to have authored an action even if it was guided or influenced by external inputs. Whether or not one knows about this influence of external inputs, or whether or not one can verbalize it, does not affect whether an action is self-generated” (Schüür and Haggard, 2011, p. 1,703). The reasoning here is straightforward: internal action-generating factors are bound to be affected by external inputs that follow from an agent’s interactions with the world; if we think this affects the self-initiated character of the actions that follow from internal causes, then we imply that self-initiated action must issue from some causally insulated part of ourselves (an “agential self”) that does not exist.

Likewise, Schlosser argues that in reason explanations the distinction between internal and external factors determining behavior is problematic. He refers to Betram Malle who compares what seems to be a situation-based explanation involving external determining factors–“Jack bought the house because it was secluded”–with an explanation involving internal causes–“Jill bought the house because she wanted privacy” (Malle, 2011; the examples are derived from Ross, 1977). Despite linguistic appearances, these explanations are not radically different. Malle’s point is not that we cannot distinguish internal from external factors; rather, it is that the first explanation merely *seems* to be a situational one. In fact, it is referring to internal factors because it is Jack’s *belief* that the house is secluded that motivated him to buy it. Still, beliefs, as factors that co-determine our choices are themselves co-constituted by our interactions with the world. These interactions are not part of the reasons for which we act, but they can nevertheless contribute to action explanation as part of the “causal history of reasons” (Malle, 2011, p. 317). Hence, “choosing and acting on the basis of reasons involves, typically, *both* internal and external factors” (Schlosser, 2014, p. 253).

These lines of reasoning, sound as they are, do not imply that *any* external factor can determine our actions without affecting the degree to which it is self-initiated. Coercion and manipulation, conscious or unconscious, clearly count as external action-determining factors, and they clearly undermine the self-initiated character of actions. The question, then, is where to draw the line; when do external influences undermine the self-initiated character of action? This is a tough question that Schlosser, Schüür, and Haggard avoid and that I will not attempt to answer fully. But there is a less tough question that Schüür, Haggard, and Schlosser avoid as well and that can be answered: what are the typical external influences involved in actions that we tend to characterize as self-initiated?

One part of the answer to this question is implied by the foregoing discussion: consciously self-initiated actions are issued by conscious deliberation or other forms of “complex integration,” but these processes themselves are operations on a diverse range of inputs, many, most, or perhaps all of which might have external origins. Belief formation is a clear case in point. But even formation of desires and preferences may be the result of subliminal priming (Schüür and Haggard, 2011, p. 1,703) or other external influences. These external influences do not undermine the self-initiated character of actions if or when they do not pre-program the outcomes of the process of complex integration and/or deliberation.

But there is another type of external influence. Sometimes external events seem to give rise to behavioral responses more or less immediately–think of my stopping automatically when a traffic light turns yellow. In such cases, it is unlikely that much conscious reflection occurs in between the trigger and the action. So, do such external triggers undermine the self-initiated character of my action? In the remainder of this section I will argue that with the notion of self-initiated action as action that follows from conscious deliberation/integration in hand, it is quite easy to distinguish between direct external triggers that are and those that are not compatible with consciously self-initiated action. The trick is to look at the way in which intentions that are the product of deliberation or integration are supposed to cause our actions.

In much of the literature on the causal (in)efficacy of intentions, causal relations are depicted as what Fred Dretske calls “triggering causes” (Dretske, 1988). This is not to say that all intentions immediately trigger actions. Most of our intentions are not aimed at actions right here and now but at actions that have to occur sometime in the future. Intentions to take the 6 o’ clock train, to have pasta for diner, to finish a paper in the upcoming week, to have a coffee in the next break, etc. are cases in point. In jargon, these are longer-term, distal, or future-directed intentions, to be distinguished from short-term, proximal, present-directed intentions (these are all relative notions, to be sure–a distal intention can be aimed at the next 5 min as well as at years from now). A distal intention is the onset of a causal “cascade” (Pacherie, 2006, 2008) of internal processes that eventually leads to an action: a distal intention will at some point in time, when the relevant circumstances to act arrive, transform into a proximal intention, which will in turn transform in the actual motor intentions that cause the appropriate bodily movements. Elisabeth Pacherie calls the process in which a distal intention is transformed into proximal and motor intentions “situational anchoring.”

How should we conceive of the process in between the formation of a distal intention and its situational anchoring? Suppose that I consciously decide that from now on I will respond to yellow traffic lights by stepping on my brake rather than on the gas (prompted, say, by a particularly expensive traffic fine). It is extremely unlikely that there is a causal chain of neural events, one triggering the other, in between my forming this intention and my acting on it. Perhaps there may be some active monitoring of the environment to determine whether the right circumstance to act occur, say whenever we are behind the steering wheel. But even then, the actual “triggering” effect of the intention to stop for yellow traffic lights is temporarily put on hold. Rather than conceiving of distal intentions as the triggering causes of our actions, it is much more plausible to conceive of them as what Dretske called “structuring causes.”^3^

The idea of a structuring cause is most easily explained by reference to an electric circuit: suppose that flipping a switch causes the light bulb to burn. Flipping the switch would then be the triggering cause. But it can only trigger the light to go on *given* that someone wired the switch and the light bulb to a power source in the appropriate way. This wiring is the structuring cause of the light bulb burning. The direct effect of this particular structuring cause is that a disposition–an if-then relation–is put in place: after the structuring cause, but not before, flipping the switch will cause the light to go on. My forming the intention to stop at yellow traffic lights from now on functions as a structuring cause. I set myself up–if my intention is indeed efficacious–to respond to external triggers (yellow traffic lights) in a specific way, immediately and quite possibly without thinking. The direct effect of my intention is not breaking but a disposition to break in response to seeing a yellow traffic light.

The idea of distal intentions as structuring, rather than triggering causes of action, explains how some direct external triggers of actions do not undermine the self-initiated character of actions. Even though I will respond automatically and unconsciously to a yellow traffic light, this can be an effect of a distal intention that has predisposed me to respond in this way. If the distal intention and hence the predisposition are the result of a process of conscious deliberation, then my stopping for a yellow traffic light may be a consciously self-initiated action, even though it is an unconscious response to an external trigger.

It is tempting to apply a similar structure to finger lifting or button pressing in Libet-style free selection tasks. As has been argued by many, starting with Dennett (1991), the efficacious intention of subjects in these experiments is not the proximal intention to lift a finger now, but the distal intention that is formed when subjects agree to lift their finger at their chosen time while keeping their eye on a clock or some other device in order to record the time of the conscious proximal intention. In all likelihood, the processing of the instructions for these experiments is conscious processes of information integration resulting in the active formation of an intention. The direct effect of the formation of this intention is a disposition to respond not to external triggers, but to internal ones (Asma, 2018, pp. 87–88). These internal triggers may, for instance, be the result of ongoing, unconscious, spontaneous fluctuations in neural activity (Schurger et al., 2012).

The emphasis on consciously formed distal intentions as the structuring causes of actions in this section is not meant to rule out the possibility that consciously formed proximal intentions can function as the direct triggers of action (Asma, 2018, pp. 87–89). This possibility might be controversial in light of the evidence against conscious proximal intentions as initiators of actions, though (then again, most of this evidence is based on research that, like Libet-style experiments, involves intentions subjects become passively aware of rather than actively formed intentions). Including the possibility of actively formed proximal intentions as the direct triggering causes of actions would not, however, alter the conclusions about the interplay of conscious and unconscious processes that I will outline in the next section. For that reason, I will not discuss this possibility further.

## The Interplay Between Conscious and Unconscious Processes in Consciously Self-Initiated Action

The upshot of our discussion so far is that consciously self-initiated actions are (1) caused by actively consciously formed intentions, not by intentions we passively become conscious of. (2) Though it is not impossible that some of these intentions are the triggering causes of our actions, many of them function as the structuring causes of actions by setting us up to become responsive to external and internal triggers. This type of human volition–consciously formed intentions as the structuring causes of actions–involves at least four stages in the process that starts with the formation of an intention and ends with the intended action in which unconscious processes play a constitutive part, *without* reducing the consciously self-initiated character of these actions. I will list and briefly discuss these stages in this section.

But not before reiterating what it is that makes an action consciously self-initiated on the current proposal. From the above discussion, one clear condition can be distilled that have to be met for this to be the case:

1. Conscious deliberation and/or conscious integration of complex information has to play a non-redundant part in the production of the intention.
  To speak meaningfully of *self*-initiated action, it might be appropriate to add a second condition. Intentions that are not person-specific while they are, in part, produced by conscious deliberation may nevertheless not be paradigmatic instances of self-initiated action if everyone arrives at the same decision. Think of travelers who read and process timetables for public transport in order to reach a decision about which train to catch. Such decisions may count as self-initiated in the literal sense that each person decides for herself. But on a “thicker” reading of “self,” according to which a self is characterized by what is distinctive of a person, the following condition may be added:
2. The outcome of this conscious process has to be person-specific–e.g., determined by a person’s biography, socialization, and/or genetic make-up–to such an extent that different people would form different, person-specific, intentions even when the inputs for their processes of intention formation are similar or identical.

This second condition is optional in the sense that it is not crucial for the rest of my argument.

These requirements for consciously self-initiated action pertain to the process of actual intention-formation only. Within the overall process that begins just before the intention is formed and ends when the intention is acted upon, this leaves four stages that might involve unconscious processes, three of which have been mentioned already in the previous section. Let me discuss these stages in chronological order, that is, in the order in which the process from forming an intention to acting on it unfolds. I will be brief about the stages that I have already discussed in the previous sections.

### Stage 1: The Inputs of Conscious Deliberation and/or Information Integration

Deliberation starts from specific beliefs, desires, preferences, etc. Information integration starts with the various bits of information, from memory, perception, thought, etc. that are going to be integrated. Many (most?) of these inputs have unconscious, sometimes external, origins. Desires, preferences, inclinations, yearnings, cravings, longings, etc. are usual things we become conscious *of –*passively. Like the examples of one’s preference for banana ice cream or the urge to move a finger in Libet’s experiments, such attitudes can be consciously picked up on, but they are usually not produced by conscious reflection. We find ourselves endowed with them. And yet, they play a crucial part in practical deliberations. The same goes for other types of inputs. Memories, perceptions, even some beliefs we have without being aware of their causal origins can all function as inputs for processes of integration/deliberation. But while they are being consciously picked up on, they are not the products of the processes of deliberation/integration they function as inputs for.

### Stage 2: Deliberation and/or Integration Resulting in the Formation of an Intention

This stage has not been discussed in the foregoing, and it may seem odd to list the one stage in which consciousness plays its essential role among the stages in which unconscious processes play a role as well. So let me discuss this stage in a bit more length than the others. This point is this: while (reflective) consciousness may be required to set a process of deliberation and/or information integration in motion, for instance, by processing the instructions of an experiment by subjects, or by “laying all options on the table” (see the previous section), the very processes of judging, weighing, or concluding need not be as fully conscious as many or most of us are inclined to think. I do not have the space (nor the argumentative materials) to argue conclusively for the unconscious nature of some processes of judging or concluding here. All I can do is cite some–very old–surprising experimental results that seem to suggest that some of the processes we think are conscious are in fact unconscious, thus making the overall second stage of “consciously forming an intention” a combination of conscious and unconscious processes.

The experimental results I have in mind are antique results from the time of introspectionist psychology. A first experiment worth mentioning was aimed at investigating the phenomenology of making very simple conscious judgments (Marbe, 1901/2012). Subjects had to lift two small weights and judge which one was heavier than the other. These highly trained subjects provided meticulous descriptions of the bodily sensations while touching and lifting the weights, but contrary to their own expectations, they found that the very act of judgment itself was not conscious. They were conscious of feeling the weights, lifting them, and of concluding that one was heavier than the other, but the very process by means of which this conclusion was reached was not consciously accessible. Or, at least, so it seemed. Because this finding was counter-intuitive and *ad hoc* hypothesis was formed: the process of judging is conscious but it happens so fast that it is missed by subjects.

In order to test this hypothesis, another experiment was set up (Watt, 1905/2011). In this experiment, subjects were shown words in response to which they had to utter an associated word quickly. This was not free association, but partially constrained association: subjects had to associate a word that was, for instance, superordinate to the shown word (e.g., wolf-animal), coordinate (wolf-lion), or subordinate (wolf-leg) (there were other options too). The experiments were “broken down” in four phases: (1) receiving the instructions (e.g., utter a coordinate association), (2) the presentation of the stimulus word, (3) searching for an appropriate association, and finally (4) uttering this association. Subjects were asked to concentrate specifically on one of these phases. It was expected that the third phase would consist of a conscious process of searching for an association–just like it was expected that Marbe’s subjects would experience a conscious process of judging. When subjects had to focus on this third phase it was expected that even though it would be a fast process–like the others–subjects would report their conscious experience of it. In fact, however, phase three was “blank.” There was no conscious experience of searching and finding an association. As it turned out, the associations just popped up in consciousness.

This information is anecdotal, and the actual findings would perhaps not count as scientific by today’s standards. And yet they should make us open to the possibility that parts of what we think of as conscious processes of integrating information or deliberating so as to form an intention to act are as not conscious, despite the fact that many of us have intuitions to the contrary.

Two further stages in which unconscious processing plays a role in the overall process from forming an intention to acting on it. These stages occur only when conscious intentions function as the structuring causes of actions (hence, they are irrelevant in cases where consciously formed intention directly triggers our actions, should these exist). The direct effect of intentions as structuring causes of actions is the formation of a behavioral disposition. The examples used in the previous section–automatically hitting the break when seeing a yellow traffic light, and a subject in Libet’s finger lifting experiment consciously taking in the experiment’s instructions–are cases in point. In these examples, the behavioral dispositions are relatively simple. But they can be more complex too. For example, I consciously decided to have lunch in the canteen at 12.30 today, thus disposing myself to respond to seeing the clock indicate 12.25 by following a complex route to the canteen, queueing for an elevator, taking the proper turns, etc., i.e., responding to the very many different relevant visual cues in appropriate ways, without giving it much thought (this is an example of an implementation intention, mentioned in the introduction; Gollwitzer, 1999; Gollwitzer and Sheeran, 2006). After forming a behavioral disposition, there are two stages that may involve unconscious processing. Let me briefly say something about both.

### Stage 3: Maintenance of a Behavioral Disposition

In between forming a disposition and acting on it, a disposition has to be maintained. In many cases, this does not require much cognitive effort. But in some cases, a disposition is maintained only because some background monitoring takes place. My intention to greet colleagues–rather than ignore them because I tend to be immersed in my own thoughts–produces a disposition to greet only if I keep track of whether people I encounter are indeed colleagues. Such a process of keeping track may be largely or wholly unconscious. Similarly, I can intend to catch the 5 o’ clock train and hence set in place a disposition to act that is maintained only because in the back of my mind I keep track of the time. If I completely lose track of the time, my disposition will disappear. Thus, in at least some cases, unconscious processes are required for intentions that function as structuring causes of our actions to actually result in corresponding actions.

### Stage 4: Situational Anchoring

Consciously produced intentions set us up to become responsive to certain external or internal triggers–they program us with specific behavioral dispositions–but the actual triggering of actions itself may well be an unconscious process. I may be conscious of the fact that I am hitting the break in response to a yellow traffic light but that does not mean that my responding is triggered, in a direct sense, by conscious reflection–I respond as in a self-produced reflex. Likewise, subjects in Libet’s finger lifting experiment have set themselves up to become responsive to subtle, internal triggers after consciously processing the instructions for the experiment. The actual triggering itself is an unconscious process. It is not unlikely that very many of our daily actions have a similar structure: making my coffee in the morning, getting on my bike to go to work, starting up my computer, etc. are very probably all actions (or sets of actions) that are the result of a conscious intentions that are their structuring causes and external or internal unconscious cues that are their triggering causes.

**Figure 1:**
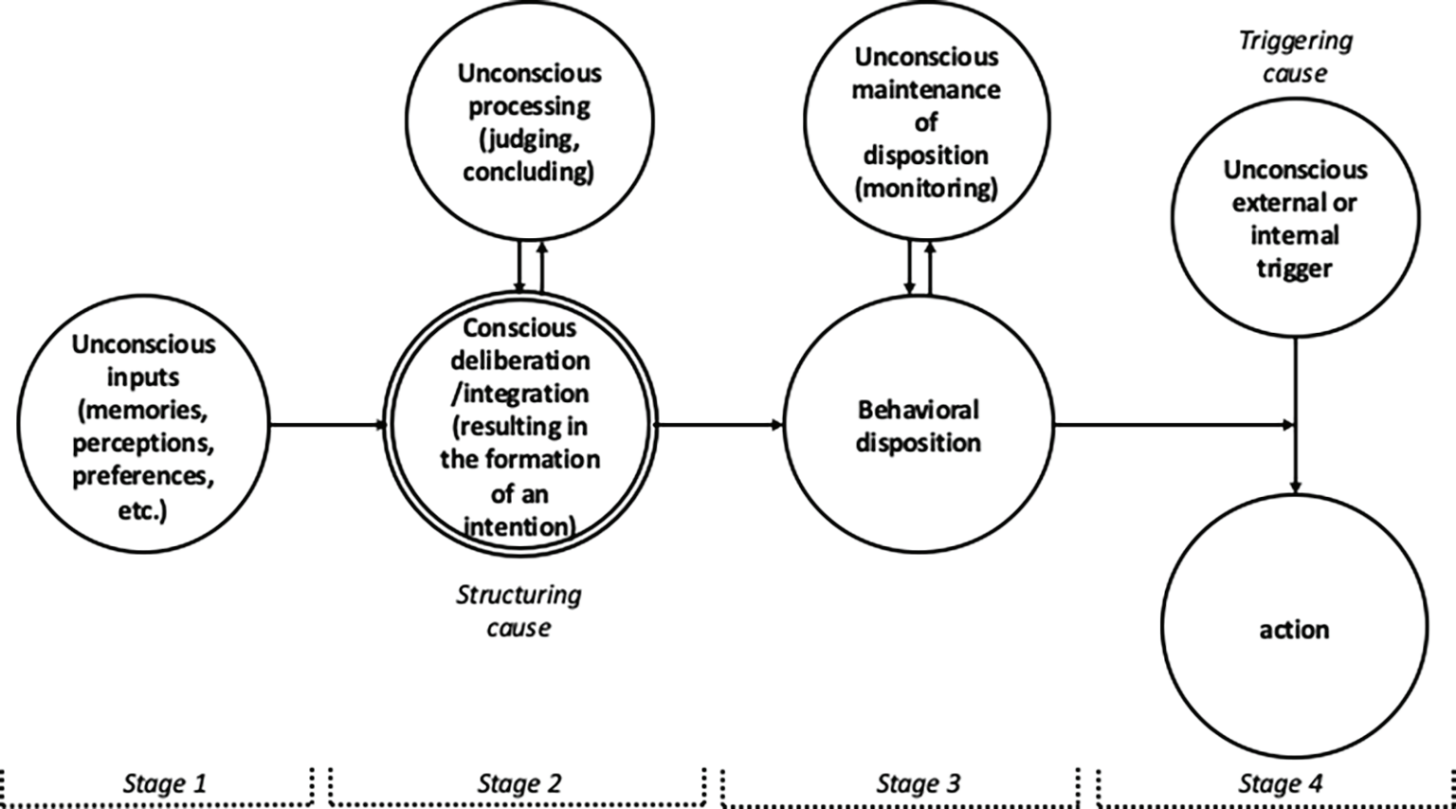
Schematic overview of the interplay between conscious and unconscious processes in consciously self-initiated actions.

The discussion of these four stages in which unconscious processing can play a role in consciously self-initiated actions is meant as nothing but a sketch of the possible unconscious contributions that may be at play in consciously self-initiated actions. Figure 1 presents a schematic overview of the resulting picture of the interplay between conscious and unconscious processes in human volition.

Which of these influences actually occur and how will differ from action to action and should be subject to empirical research. The idea here is merely to give a more structured and more detailed picture of the interplay between conscious and unconscious processes in human volition than is provided by Baumeister and Masicampo’s (2010) remark *that* such interplay is likely to play an important role in consciously initiated actions. The role of unconscious processes as depicted in Figure 1 is possibly more extensive than Baumeister and Masicampo intended. But, the really important thing to note is that despite the possibility that unconscious processes play a significant role in all four stages, if the process of conscious deliberation/complex integration meets the two requirements mentioned at the beginning of this section, the ensuing action will still be genuinely consciously self-initiated.

## Conclusions

The aim of this article was to argue that two conceptual distinctions allow for a more detailed picture of the interplay between conscious and unconscious processes in consciously self-initiated action. First, I have argued that the distinction between consciously formed intentions and intentions we become conscious of allows us to see that *only* the former is relevant to the notion of consciously self-initiated actions. This ties in with existing philosophical and scientific criticism of free selection paradigms and the suggestion that self-initiated action should involve deliberation and/or complex integration of diverse inputs.

Next, I argued that consciously formed intentions as the initiators of actions allow for a more complex and realistic picture of volitional processes when we distinguish between structuring and triggering causes. At least in many instances, consciously formed intentions are the structuring causes, rather than the triggering causes of our actions. This allows for the possibility that actions are unconsciously triggered and can yet count as consciously self-initiated because the triggering depends on there being a behavioral disposition that is (structurally) caused by consciously formed intentions.

Finally, I argued that just like consciously self-initiated actions can be unconsciously triggered, there are three earlier stages in the process that starts with conscious intention formation and ends with acting on such an intention in which unconscious processing plays a role: the input stage of processes of conscious deliberation and/or integration; the stage of deliberation/integration in which some processes of judging, weighing, or concluding many not be conscious; and the process in which the behavioral disposition that is the result of a consciously formed intention is maintained.

It is in the nature of conceptual distinctions that we cannot draw empirical conclusions from them; the resulting picture of the interplay of conscious and unconscious processes in human volition is schematic and very general. It is meant to function as a framework for interpreting the results of existing experiments and, possibly, for setting up new ones.

## Author Contributions

The author confirms being the sole contributor of this work and has approved it for publication.

### Conflict of Interest Statement

The author declares that the research was conducted in the absence of any commercial or financial relationships that could be construed as a potential conflict of interest.

## Footnotes

^1^Thanks to an anonymous referee for pointing this out.

^2^I would like to thank an anonymous referee for pressing me on this.

^3^Wu (2011) uses this notion in a similar way in a slightly different context. I have defended it earlier (Slors, 2015) in connection with Libet, Wegner, and other neuroscientific attacks on the conscious will. Pacherie (2015) agrees that the notion of structuring causes is a fruitful one in the context of conscious agency and refers to both publications. I am not aware of other uses of this concept in this context. Given its intuitive plausibility and usefulness in this context, it is not unlikely that I am missing references.
